# Changes to Airborne Pollen Counts across Europe

**DOI:** 10.1371/journal.pone.0034076

**Published:** 2012-04-13

**Authors:** Chiara Ziello, Tim H. Sparks, Nicole Estrella, Jordina Belmonte, Karl C. Bergmann, Edith Bucher, Maria Antonia Brighetti, Athanasios Damialis, Monique Detandt, Carmen Galán, Regula Gehrig, Lukasz Grewling, Adela M. Gutiérrez Bustillo, Margrét Hallsdóttir, Marie-Claire Kockhans-Bieda, Concepción De Linares, Dorota Myszkowska, Anna Pàldy, Adriana Sánchez, Matthew Smith, Michel Thibaudon, Alessandro Travaglini, Agnieszka Uruska, Rosa M. Valencia-Barrera, Despoina Vokou, Reinhard Wachter, Letty A. de Weger, Annette Menzel

**Affiliations:** 1 Chair of Ecoclimatology, Technische Universität München, Freising-Weihenstephan, Germany; 2 Institute for Advanced Study, Technische Universität München, Garching, Germany; 3 Institute of Zoology, Poznań University of Life Sciences, Poznań, Poland; 4 Botany Unit and Institute of Environmental Science and Technology (ICTA), Universitat Autònoma de Barcelona, Bellaterra, Spain; 5 Allergie-Centrum-Charité, Charité-Universitätsmedizin Berlin, Berlin, Germany; 6 Laboratorio Biologico, Agenzia provinciale per l'ambiente, Laives (BZ), Italy; 7 Dipartimento di Biologia, Università di Roma “Tor Vergata”, Roma, Italy; 8 Department of Ecology, School of Biology, Aristotle University, Thessaloniki, Greece; 9 Section Mycology and Aerobiology, Scientific Institute of Public Health, Brussels, Belgium; 10 Department of Botany, Ecology and Plant Physiology, University of Córdoba, Córdoba, Spain; 11 Federal Office of Meteorology and Climatology MeteoSwiss, Zurich, Switzerland; 12 Laboratory of Aeropalynology, Adam Mickiewicz University, Poznań, Poland; 13 Department of Plant Biology II, Complutense University of Madrid, Madrid, Spain; 14 Icelandic Institute of Natural History, Gardabær, Iceland; 15 Station d'Aérobiologie, Centre Hospitalier de Luxembourg, Luxembourg, Luxembourg; 16 Department of Clinical and Environmental Allergology, Jagiellonian University, Medical College, Kraków, Poland; 17 Department of Biology, National Institute of Environmental Health, Budapest, Hungary; 18 National Pollen and Aerobiology Research Unit, University of Worcester, Worcester, UK; 19 Réseau National de Surveillance Aérobiologique, Brussieu, France; 20 Laboratory of Palaeoecology and Archaeobotany, Gdańsk University, Gdańsk, Poland; 21 Department of Biodiversity and Environmental Management, Universidad de León, León, Spain; 22 Polleninformationsdienst Deutschland (PID), Ganderkesee, Germany; 23 Department of Pulmonology, Leiden University Medical Center, Leiden, The Netherlands; University of Oxford, United Kingdom

## Abstract

A progressive global increase in the burden of allergic diseases has affected the industrialized world over the last half century and has been reported in the literature. The clinical evidence reveals a general increase in both incidence and prevalence of respiratory diseases, such as allergic rhinitis (common hay fever) and asthma. Such phenomena may be related not only to air pollution and changes in lifestyle, but also to an actual increase in airborne quantities of allergenic pollen. Experimental enhancements of carbon dioxide (CO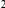) have demonstrated changes in pollen amount and allergenicity, but this has rarely been shown in the wider environment. The present analysis of a continental-scale pollen data set reveals an increasing trend in the yearly amount of airborne pollen for many taxa in Europe, which is more pronounced in urban than semi-rural/rural areas. Climate change may contribute to these changes, however increased temperatures do not appear to be a major influencing factor. Instead, we suggest the anthropogenic rise of atmospheric CO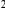 levels may be influential.

## Introduction

Many factors have been proposed to explain the 20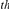 century increase [1]–[4] in the burden of allergic respiratory diseases, although the causes are still not fully understood [5]. For example, air pollution can influence both allergens and allergic subjects in many ways, making the former more potent and increasing the immune reaction of the latter [6]. However, these phenomena are insufficient to explain completely the increased rate of allergic diseases in humans [6].

Plant phenology, the timing of life cycle events in vegetation (e.g. budburst, flowering), is generally sensitive to temperature [7], [8]. If not water-limited, it has responded strongly to global warming [7], [9]. Hence, it can be reasonably supposed that global change also affects pollen timing and production [10], [11]. These may contribute to the increasing trend in allergic diseases. However, single studies on pollen quantities in recent years have been inconclusive, e.g. inconsistent trends for five pollen types at five sites in Western Europe [12], or a more consistent increase for many taxa in Thessaloniki, Greece [13].

**Figure 1:**
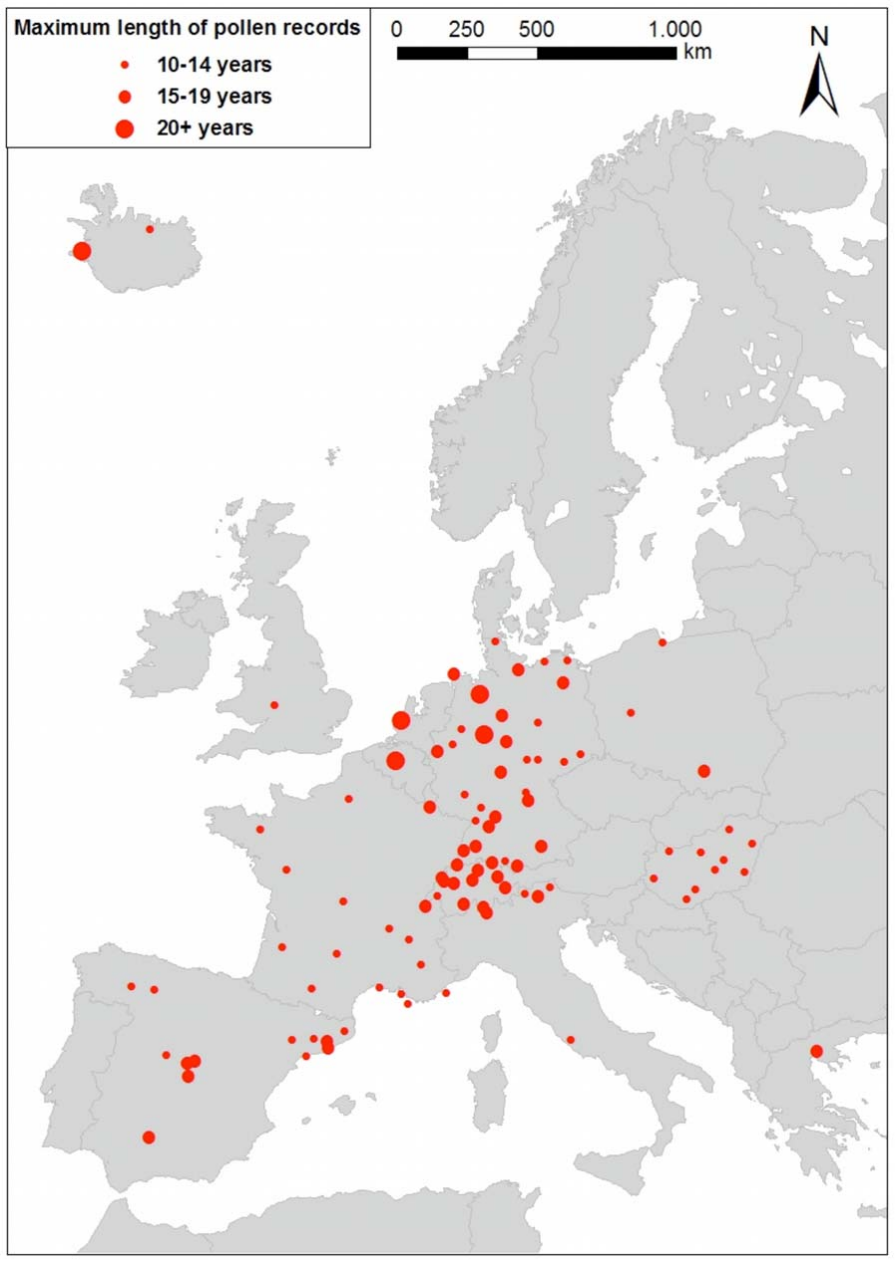
Locations of pollen sites. Each station has been indicated by a red circle. Symbol sizes are proportional to the temporal length of the local longest pollen record.

Current aeropalynological research uses a number of different indicators to describe the pollen season (e.g., start and end dates, daily concentrations, timing of peak production). Past study results may have been influenced by the choice of indicator used [14]. In the present analysis of 1221 European pollen time series at 97 stations (see Fig. 1), we focus on yearly trends of the annual pollen index (API), a quantity universally defined as the sum of average daily pollen concentrations over the year. The trends of API at each monitored location were normalized by the respective mean API. This normalized index allows a comparison across different provenances and microclimates within the large geographic range of species in Europe. Moreover, using this normalization, the different methodologies used to measure daily pollen concentrations are less likely to influence calculation or detection of temporal trends.

## Results and Discussion

### Trends in pollen counts

**Figure 2:**
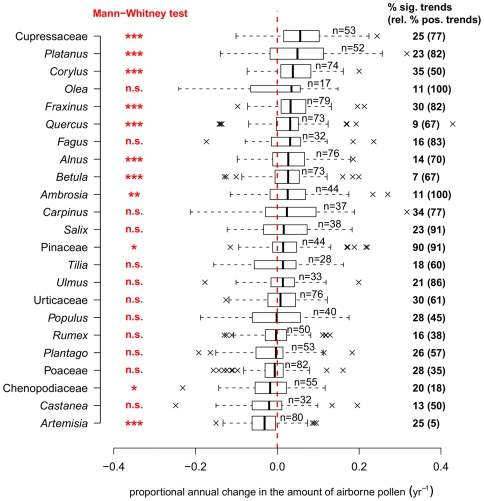
Trends of annual pollen index (API) by species. Boxplots show the proportional annual change of yearly pollen sums for the 23 pollen taxa analyzed (reasons for selection given in the main text). Medians are significantly different from zero (Mann-Whitney test, * : 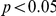, ** : 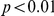, *** : 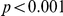, n.s.: 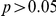) for 11 taxa. On the right, the percentages of significant trends are indicated for each taxon (of which the percentages of positive trends are given in parentheses). The height of the boxplot is related to sample size, taxa are arranged in decreasing order of their medians.

**Figure 3:**
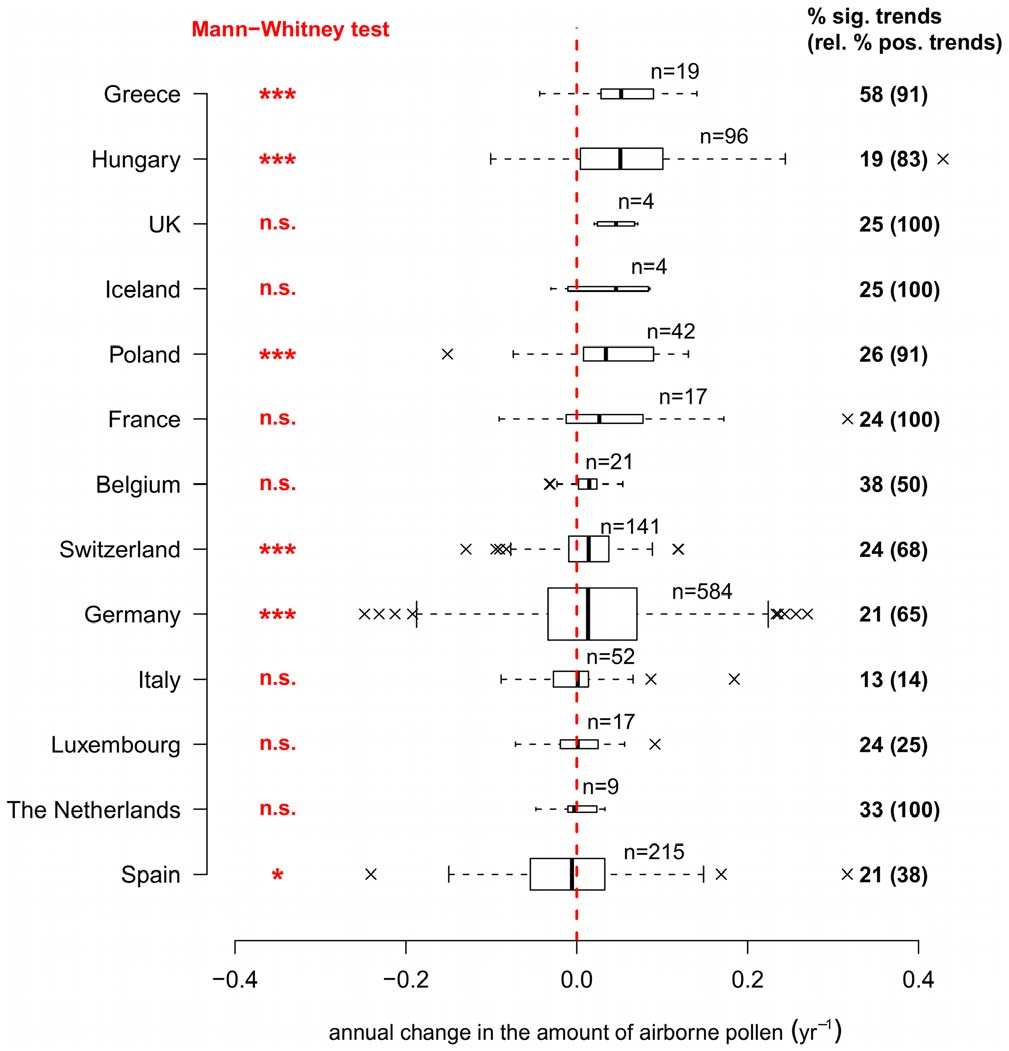
API trends by country. Boxplots show the proportional annual change of yearly pollen sums for 13 countries. Medians are significantly different from zero (Mann-Whitney test, * : 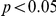, ** : 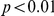, *** : 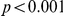, n.s.: 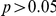) for six countries. On the right, the percentages of significant trends are indicated for each country (of which the percentages of positive trends are given in parentheses). The height of the boxplot is related to sample size, countries are arranged in decreasing order of their medians.

Analyses showed that 724 (59%) APIs increased and 497 (41%) decreased. 271 (22% of the total) were statistically significant (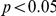), among which 171 (14% of the total) increased and 100 (8% of the total) decreased. In Fig. 2, annual changes in API are summarized for 23 families or genera chosen amongst important allergenic pollen types according to the sensitization and allergic symptoms of people living in specific regions, (e.g., *Alnus*, *Ambrosia*, *Artemisia*, *Betula*, *Corylus*, Cupressaceae, *Olea*, Poaceae), or from constantly important land-uses (e.g., *Fraxinus*, *Platanus*, *Carpinus*, *Castanea*, Pinaceae, *Plantago*, *Quercus*, *Rumex*). For nine taxa, all with highly allergenic pollen, the indices increased significantly (Mann-Whitney test, 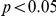), while only two taxa decreased significantly. API trends from tree species were, in general, larger than those from herbs and shrubs. In recent years, some tree taxa (e.g., Cupressaceae) have been extensively used as ornamental plants in cities, and hence their pollen trends could have been positively affected by urban planning. However, land-use changes in general (e.g., afforestation) may be too slow to explain increasing API in trees. The significant decreases for Chenopodiaceae and *Artemisia* could be possibly explained by intensification of weed control and less agricultural land set-aside in the context of increasing bioenergy demand. Analysis by countries (Fig. 3) also reveals a general increase in API, with 11 of 13 countries having median changes greater than zero, significant for five countries. The significant decrease for Spain, although the median trend is close to zero, is somewhat surprising, especially in light of a recent study reporting an increase in grass pollen in southern Spain [15]. Our result for Spain is unlikely to be biased by a small sample size (215 series analyzed). Furthermore, our Spanish data cover a wider geographic range with varied water availability which may be more influential on API, particularly of grasses, in Spain compared to more northern countries.

A large variability in the API trends is evident, shown by the presence of large outliers in the boxplots of Fig. 2 and Fig. 3. Outliers may be caused by rainy weather during the pollen season reducing annual totals, favourable (warm and dry) weather, episodes of long-range transport, inherent inter-annual variation of pollen production (years of massive and synchronized pollen production by plants, the so-called masting behaviour), re-suspension phenomena (winds raising deposited pollen in the lower atmosphere), and abrupt changes in species density by local land management. Further research is needed to identify the relative importance of each of these factors.

### Considered drivers

**Figure 4:**
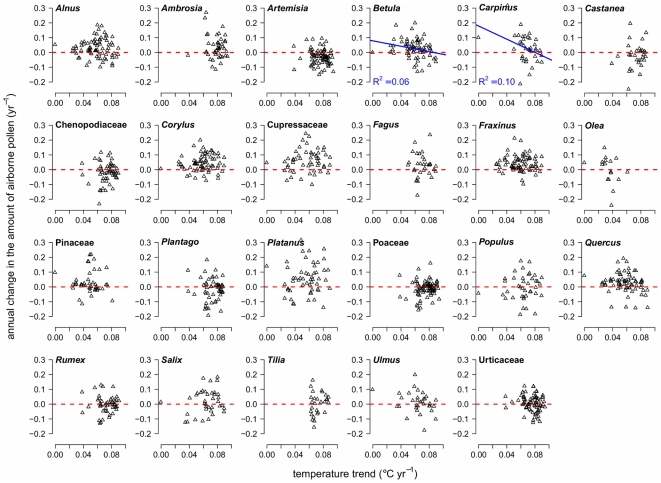
API trends against temperature trends by species. Proportional annual change of yearly pollen sums was plotted against local temperature trends for 23 pollen taxa. Temperature trends were calculated for each location for the mean temperature of two seasons, January to April (associated with the flowering of *Alnus*, *Betula*, *Carpinus*, *Corylus*, Cupressaceae, *Fagus*, *Fraxinus*, *Olea*, Pinaceae, *Platanus*, *Populus*, *Quercus*, *Salix*, and *Ulmus*) or April to August (related to *Ambrosia*, *Artemisia*, *Castanea*, Chenopodiaceae, *Plantago*, Poaceae, *Rumex*, *Tilia*, and *Urtica*), over the years 1977–2009. A regression line has been superimposed for *Betula* and *Carpinus*, the only statistically significant relationships.

In an attempt to identify the causes of pollen increases, we tested the correlation between trends in API and trends of local mean temperature. As shown in Fig. 4, there was little evidence of correlation. This could be due to not matching exactly the lengths (10 to 28 years) and gaps of the pollen series with lengths of the temperature series (33 years). Only trends in *Betula* and *Carpinus* pollen amounts showed a significant but weak correlation, which was negative. *Betula* predominantly grows in mid to high latitudes at lower temperatures, and it has been hypothesized that an increase in temperature could limit its physiological performance [16], including the production of pollen [17]. The significant negative correlation between *Betula* pollen and temperature trends seems to support such a hypothesis.

**Figure 5:**
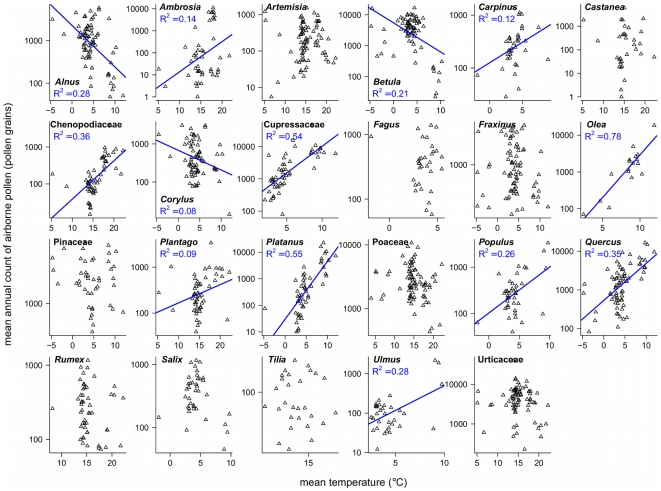
Mean API against mean local temperature. Log-scaled mean annual sum of airborne pollen was plotted against local mean temperature for 23 pollen taxa. Mean temperatures were calculated for two periods, January to April (associated with the flowering of *Alnus*, *Betula*, *Carpinus*, *Corylus*, Cupressaceae, *Fagus*, *Fraxinus*, *Olea*, Pinaceae, *Platanus*, *Populus*, *Quercus*, *Salix*, and *Ulmus*) or April to August (related to *Ambrosia*, *Artemisia*, *Castanea*, Chenopodiaceae, *Plantago*, Poaceae, *Rumex*, *Tilia*, and *Urtica*), over the period 1977–2009. Only significant regression lines are shown.

Because consistent correlations between API trends and local temperature trends could not be demonstrated, we tested instead general relationships between mean API and mean local temperature. These are shown in Fig. 5. For many species regression lines were statistically significant. Except for three tree species this relationship was positive (i.e., more pollen at higher temperatures, indicating warmer southern sites or urban sites). In contrast, *Alnus*, *Betula*, and *Corylus* are tree genera more associated with high latitudes and low temperatures, thus their negative correlation of API with temperature could reflect the limited presence of these species at warmer sites. However, variation in the density of species will influence any API-temperature relationship. Therefore, we used the European Forest Data Centre (EFDAC) data set, which includes density information. These maps, the only ones available for Europe, display the species distribution in ha of tree cover per species at a 1 km resolution. For each taxa the respective tree species covers were determined by GIS within a radius of 10 km around each pollen station. Unfortunately, according to this data set, the majority of pollen sites was characterized by a complete absence of trees, due to the forest/non-forest GIS layer used that excluded human settlements and agricultural land. Thus, no hypothesis of linking API trends with temperature and density could be tested.

**Figure 6:**
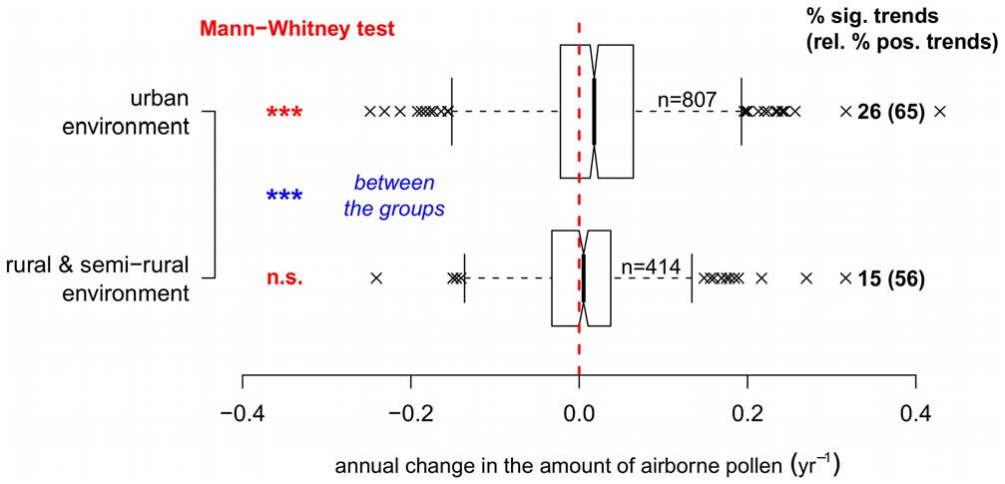
API trends by environment type. Boxplots show the proportional annual change of yearly pollen sums for different environments. Mann-Whitney tests show a significant increase (median different from zero, 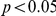) of airborne pollen in urban environments. The notches are calculated as 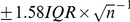 and the height of each boxplot is related to sample size. On the right, the percentages of significant trends are indicated for each type of environment (of which the percentages of positive trends are given in parentheses).

The environment in which the pollen was measured may influence results. In Fig. 6, boxplots of observed pollen trends in urban and in semi-rural/rural areas indicate a significant difference between these environments as well as an overall increase in pollen at urban sites (Mann-Whitney tests, 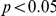). Urban environments are characterized not only by the “heat island” effect, but also by high levels of pollutants, such as NO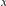, VOCs or particulates. Furthermore, higher atmospheric CO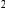 concentrations are known to cause a general increase in vegetation biomass (at least temporarily), an increase in pollen production [18]–[22], also shown in Free-Air CO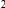 Enrichment (FACE) experiments [23] and, probably, pollen allergenicity [24]. Therefore, it can be inferred that higher levels of CO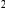, typical of urban areas, may cause a greater presence of airborne pollen in this environment. Lower tropospheric ozone (O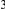) levels also characterize urban environments, due to higher concentration of nitrogen oxide (NO), which is involved in the breakdown of O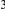. Because the effect of O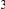 is to inhibit plant development [23], enhanced plant growth in urban areas has already been reported [25].

In addition, we tested the correlation of API and API trends (also by taxa) with latitude, longitude and altitude a.s.l. of the pollen stations, attempting to find geographical patterns in the observed changes in pollen amounts. However, no specific pattern could be detected, suggesting that regional differences in behaviour were small relative to background variability. Thus, possible biogeographical differences in behaviour are unlikely to have masked the overall reported trends here. We also tested for differences in API and its trends associated with specific plant traits, such as late-successional (e.g., *Fagus*) against early-successional taxa (e.g., *Betula*). Also in this case, no significant result could be found.

A delayed or missing fulfillment of the chilling requirement of plants for bud burst and thus flowering could play a key role under future scenarios of increasing winter temperatures. Even if not directly connected to the production of pollen, which is more sensitive to water availability, pre-flowering weather conditions (especially for herbs and grasses) [26], or weather conditions in the year preceding flowering (for some trees, such as birch) [27], chilling temperatures may influence the timing of flowering in trees [28], [29]. A late or missing fulfillment of such a requirement may delay or, in the worst case, prevent flowering events, as hypothesized for fruit and nut trees [30], [31]. As a consequence, length and intensity of the pollen season could be notably reduced, especially for species native to the Mediterranean area, where the greatest changes in winter temperature are expected for Europe [32].

### Conclusions

Despite the lack of unequivocally identified drivers, it is evident that there is currently a clear tendency towards an increase in atmospheric pollen, including highly allergenic taxa. These trends could not be attributed to rising temperatures, but may be influenced by the anthropogenic increase of the greenhouse gas CO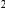 as (experimental) studies suggest [18]–[24]. More research is needed in this area because a further worldwide increase in atmospheric CO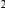 is projected, e.g. by IPCC [32]. These changes may result in further increases in pollen amounts leading, in turn, to a greater exposure of humans to pollen allergens, with potentially serious consequences for public health.

## Materials and Methods

**Figure 7:**
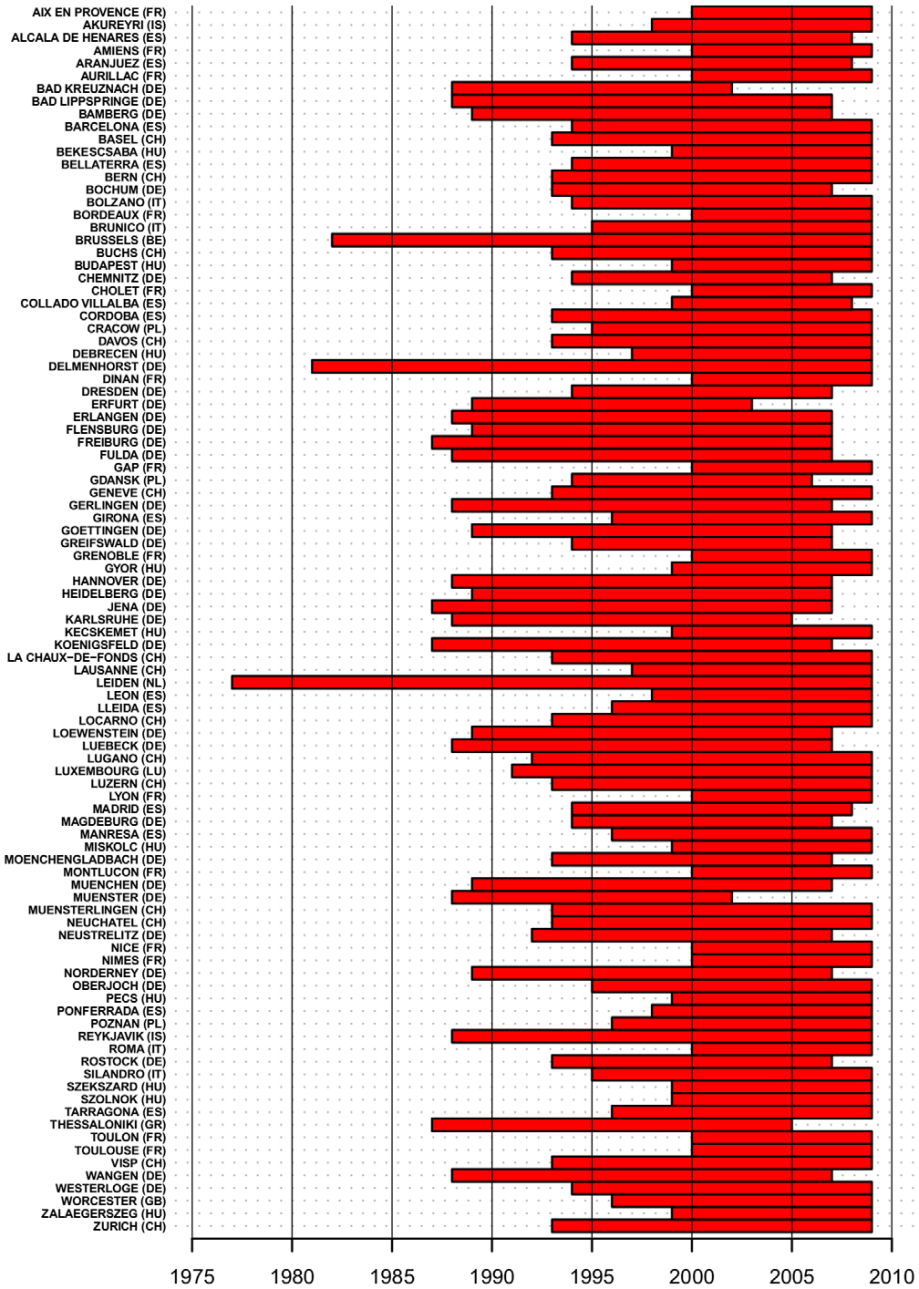
Maximum duration of pollen series by location. The local longest monitored period is shown as a red bar for each of the 97 locations considered. Missing years, occurring in few cases, have been omitted for clarity.

The analyzed data set consists of 1221 pollen time series at 97 locations in 13 European countries from 23 pollen taxa (see Fig. 1). Not every species was present in every location. Series length ranged from 10 to 28 years in the period 1977 to 2009. In Fig. 7, the longest local monitored periods are reported (short time gaps, occurring for few locations, have been omitted for clarity). Temporal trends of API were calculated as slopes from linear regression on time (years) and were normalized (i.e., converted to proportional change per year) by dividing by the mean local API.

Trends in temperature were calculated over the years 1977–2009 for two seasons, January to April or April to August, associated with different species according to their flowering period. For each pollen station, temperature data of the respective grid cell of the ENSEMBLE project data were used [33]. The temperature data from the ENSEMBLE data set, available at www.ensembles-eu.org, are based on a geographical grid of resolution 0.5 degrees latitude 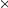
0.5 degrees longitude.

The statistical software R version 2.11.1 was used for both statistical analyses and to generate figures [34].
